# Supplementary figures and images for: Different expression pattern of flowering pathway genes contribute to male or female organ development during floral transition in the monoecious weed Ambrosia artemisiifolia L. (Asteraceae)
Source: PeerJ. 2019 Oct 4;7:e7421. doi: 10.7717/peerj.7421 (PMC6779118; doi:10.7717/peerj.7421)

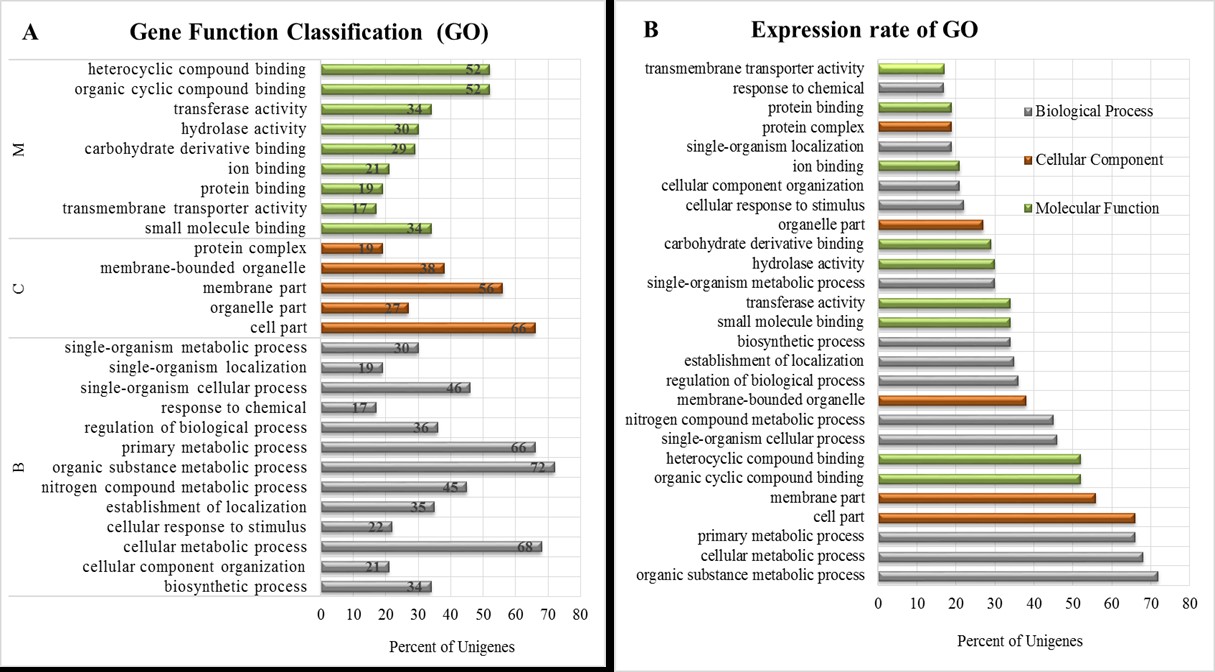

Supplement: Supplemental Information 1 — (A) Expression pattern of transcript products within the category of (C) cellular component, (M) molecular function, and (B) biological process sub-ontologies. (B) Sub ontologies were arranged according to Expression rate. The most intense gene expressions were found to be in metabolic processes, catalytic and binding activity ontologies. [file peerj-07-7421-s001.jpg]

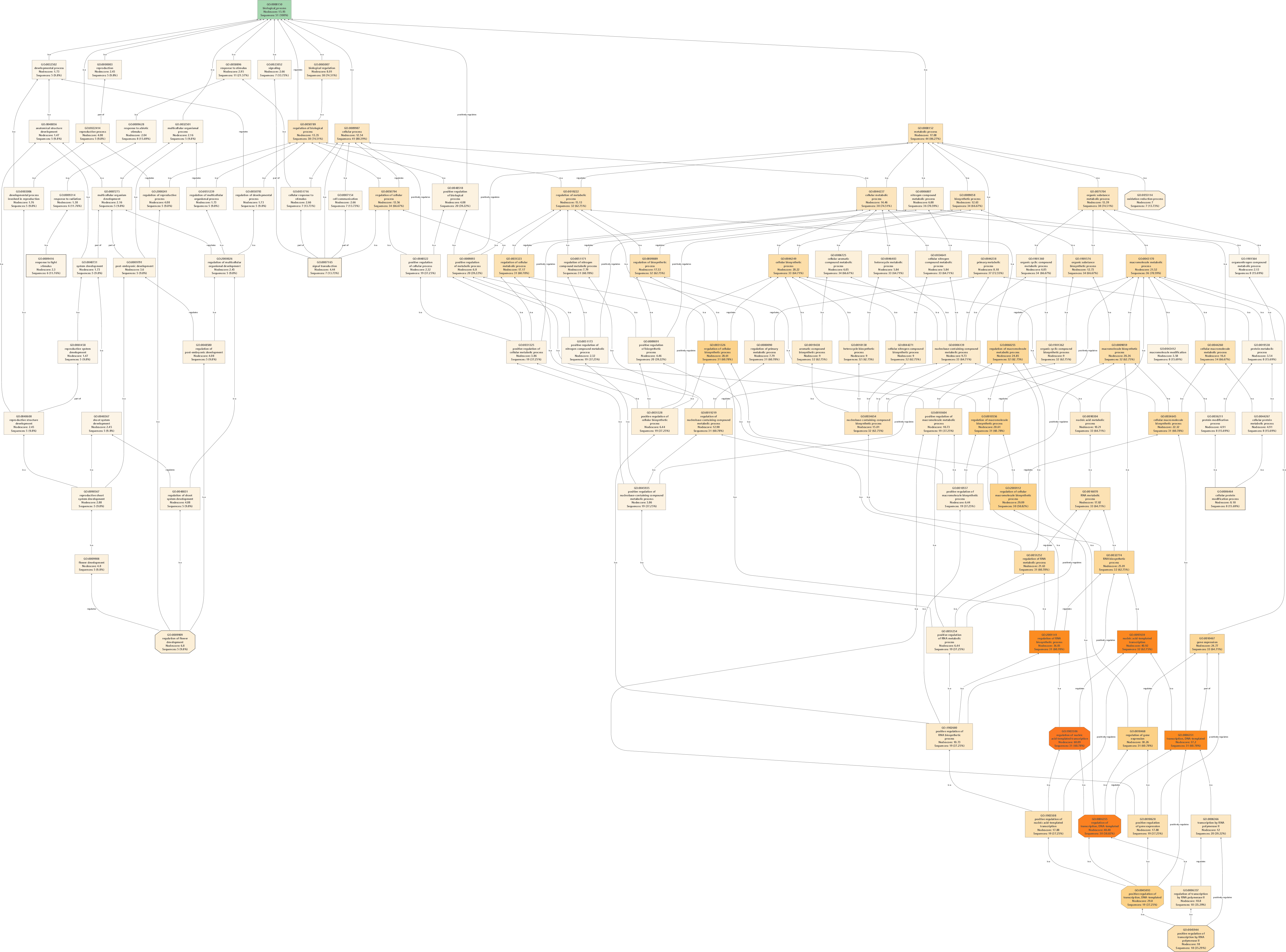

Supplement: Supplemental Information 2 — GO graph performed based on GO table of 80 discussed genes. [file peerj-07-7421-s002.png]

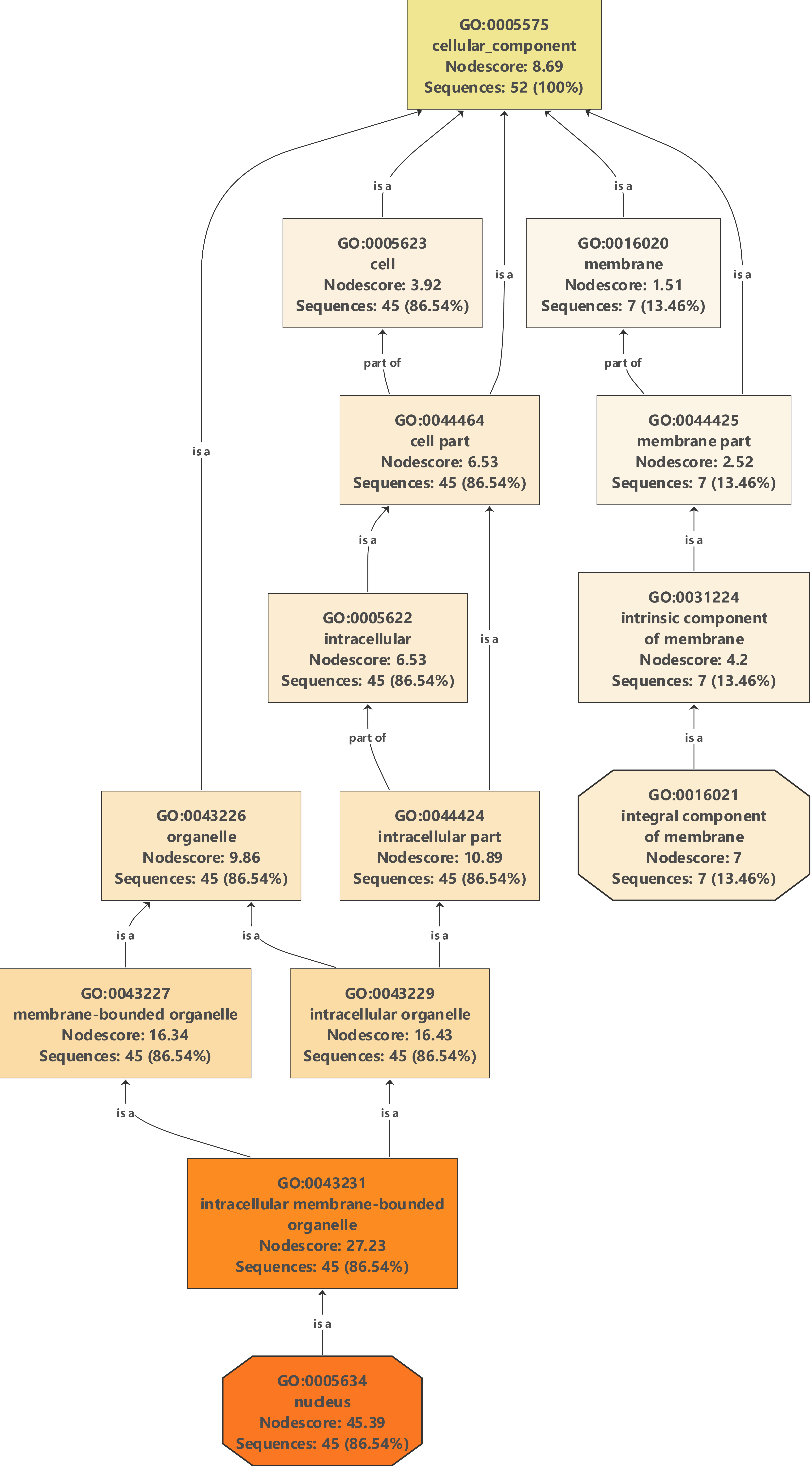

Supplement: Supplemental Information 3 — GO graph performed based on GO table of 80 discussed genes. [file peerj-07-7421-s003.png]

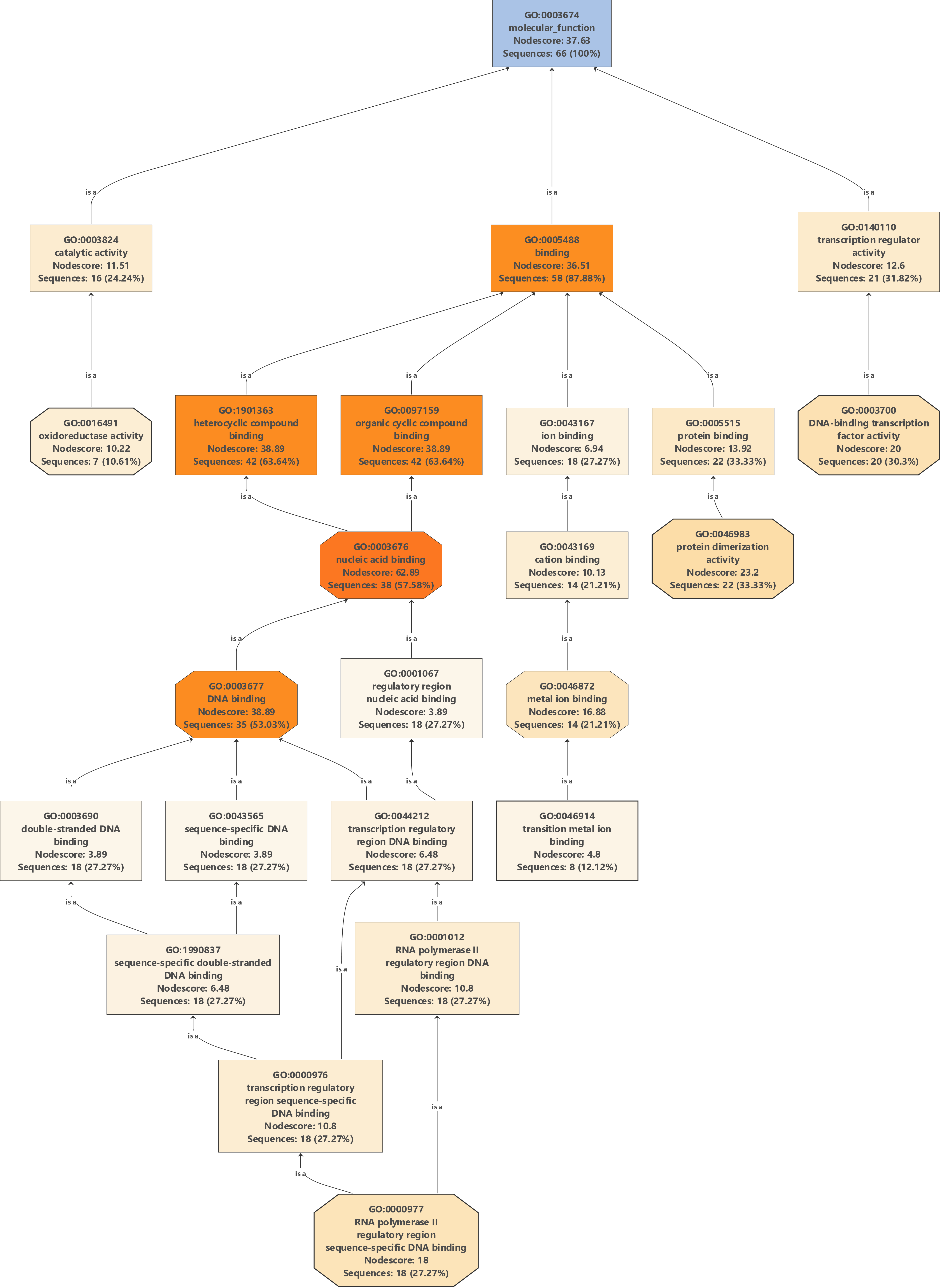

Supplement: Supplemental Information 4 — GO graph performed based on GO table of 80 discussed genes. [file peerj-07-7421-s004.png]

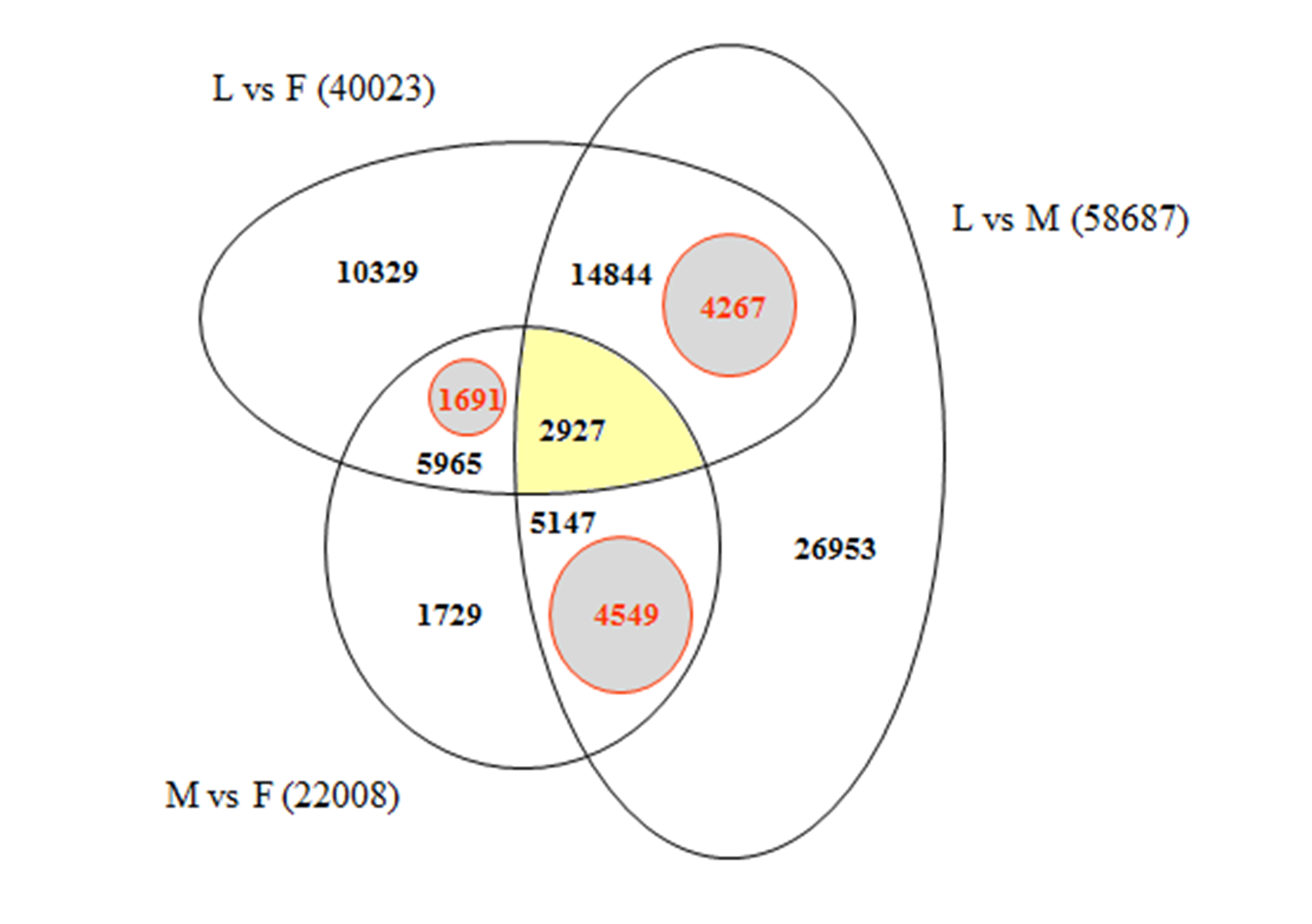

Supplement: Supplemental Information 11 [file peerj-07-7421-s011.zip › SQL/Fig5label.png]
