# Supplementary material for: Different expression pattern of flowering pathway genes contribute to male or female organ development during floral transition in the monoecious weed Ambrosia artemisiifolia L. (Asteraceae)
Source: PeerJ. 2019 Oct 4;7:e7421. doi: 10.7717/peerj.7421 (PMC6779118; doi:10.7717/peerj.7421)
Supplement: Supplemental Information 5 [file peerj-07-7421-s005.docx]

| **Sample** | **Average weight (mg)** |
| --- | --- |
| Male flower stage 1 (Figure1 M A) | 0,3 |
| Male flower stage 2 (Figure1 M B) | 2,1 |
| Male flower stage 3 (Figure1 M C) | 8 |
| Male flower stage 4 (Figure1 M D1) | 0,9 |
| Male flower stage 5 (Figure1 M D2) | 1,8 |
| Male flower stage 6 (Figure1 M D3) | 3 |
| Male flower stage 7 (Figure1 M D4) | 8,6 |
| Female flower stage 1 (Figure1 F a) | 0,1 |
| Female flower stage 2 (Figure1 F b) | 0,2 |
| Female flower stage 3 (Figure1 F c) | 0,4 |
| Female flower stage 4 (Figure1 F d) | 0,5 |
| Female flower stage 5 (Figure1 F e) | 0,7 |
| Female flower stage 6 (Figure1 F f) | 0,9 |
| Female flower stage 7 (Figure1 F g) | 1 |
| Female flower stage 8 (Figure1 F h) | 1,2 |
| Female flower stage 9 (Figure1 F i) | 1,8 |
