# Supplementary material for: Different expression pattern of flowering pathway genes contribute to male or female organ development during floral transition in the monoecious weed Ambrosia artemisiifolia L. (Asteraceae)
Source: PeerJ. 2019 Oct 4;7:e7421. doi: 10.7717/peerj.7421 (PMC6779118; doi:10.7717/peerj.7421)
Supplement: Supplemental Information 6 — Validation of F, M, and L transcriptomes were performed by using Q-RT-PCR. Genes expressed characteristically in different transcriptomes were selected based on in silico analysis. Primers used for qPCR were designed based on in silico predicted sequences. [file peerj-07-7421-s006.docx]

| **Genes** | **Forward primer sequences** | **Reverse primer sequences** |
| --- | --- | --- |
| *LAP6* | CGAGGTTACGAAGAAGAATGC | ATGGATTTTGGGAATGTGAGTAG |
| *STIG1* | CATCCCATAGGCACAAGACTC | AAGGGAAGTGTTTTGATGTGTC |
| *SUPERMAN* | CAAATGGGAAAGCAACAACTAC | AAGAGAGGGTGGTGAAGACTG |
| TCP12 | AGCTTTTGGAATTCACTTGAGTC | GAATAGTCACCAACCCAATGC |
| PI | AGAACACAAACAACAGGCAAG | GTGTTAGGGCTGCAATACTCA |
