# Supplementary material for: Different expression pattern of flowering pathway genes contribute to male or female organ development during floral transition in the monoecious weed Ambrosia artemisiifolia L. (Asteraceae)
Source: PeerJ. 2019 Oct 4;7:e7421. doi: 10.7717/peerj.7421 (PMC6779118; doi:10.7717/peerj.7421)
Supplement: Supplemental Information 7 [file peerj-07-7421-s007.docx]

|  | 1M | 2M | 1F | 2F | L | F | M |
| --- | --- | --- | --- | --- | --- | --- | --- |
| Ref Gene | RPKM | RPKM | RPKM | RPKM | RPKM | RPKM | RPKM |
| GAPDH | 4795.676 | 4914.222 | 3321.383 | 2480.102 | 3611.886 | 2676.673 | 3565.147 |
| TUA | 539.2924 | 584.5263 | 756.7592 | 884.4905 | 724.1057 | 41.83522 | 183.3697 |
| TUB | 805.3833 | 721.7192 | 811.3353 | 348.1219 | 19.98569 | 160.036 | 263.883 |
