# Supplementary material for: Different expression pattern of flowering pathway genes contribute to male or female organ development during floral transition in the monoecious weed Ambrosia artemisiifolia L. (Asteraceae)
Source: PeerJ. 2019 Oct 4;7:e7421. doi: 10.7717/peerj.7421 (PMC6779118; doi:10.7717/peerj.7421)
Supplement: Supplemental Information 8 — Libraries were generated from gender specific RNA pools. Nominations: male flowers for eight developmental stages (M), young leaves (L), and female flowers for eight developmental phases (F), classified into early (1F) and late (2F) stages. In order to represent both vegetative and generative transcriptomes a combined library from M, L, and F samples was generated. In order to analyze expression in detail induring pistillate organogenesis the nine female flower phenophases were classified into two libraries and aligned to the A. artemisiifolia reference transcriptome. [file peerj-07-7421-s008.docx]

|  | **Sample** | **M** | **L** | **F** | **1F** | | **2F** | |
| --- | --- | --- | --- | --- | --- | --- | --- | --- |
| **Sequencing** | Illumina platform | HiSeq2000 | HiSeq2000 | HiSeq2000 | NextSeq500 | | NextSeq500 | |
|  | Number of raw reads | 24,110,256 | 24,330,693 | 23,264,636 | 39,664,366 | | 37,127,852 | |
|  | Number of clean reads | 18,472,374 | 17,435,976 | 15,290,201 | 36,491,216 | | 34,157,623 | |
|  | Average read length (bases) | 2*100 | 2*100 | 2*100 | 2*80 | | 2*80 | |
| **Accession number** | NCBI SRA | SRR3995704 | SRR3995705 | SRR3995703 | SRR5965731 | | SRR5965732 | |
|  | NCBI TSA | GEZL00000000 | | | GFWB00000000 | | GFWS00000000 | |
| **Assembly** | **strategy** | **Combined reads for de novo assembly (used as reference)** | | | **de novo** | **ref. guided** | **de novo** | **ref. guided** |
|  | assembler | Trinity | | | Trinity | Bowtie2 | Trinity | Bowtie2 |
|  | total assembled reads | 1,377,646 | | | 20,421,456 | 33,108,438 | 18,032,538 | 30,611,092 |
|  | Number of contigs | 229,116 | | | 109,452 | 147,457 | 97,239 | 141,588 |
|  | Range of contig lenght | 224 -14,368 | | | 201 -8,854 | 210 - 14,375 | 201 -6,882 | 2015 - 14,370 |
|  | N50 lenght | 774 | | | 669.7 | 697.5 | 635 | 626.9 |
