# Supplementary material for: Different expression pattern of flowering pathway genes contribute to male or female organ development during floral transition in the monoecious weed Ambrosia artemisiifolia L. (Asteraceae)
Source: PeerJ. 2019 Oct 4;7:e7421. doi: 10.7717/peerj.7421 (PMC6779118; doi:10.7717/peerj.7421)
Supplement: Supplemental Information 9 [file peerj-07-7421-s009.docx]

| **Gene homolog** | **RPKM value (L)** | **RPKM value (M)** | **RPKM value (F)** | **Function** | **References** |
| --- | --- | --- | --- | --- | --- |
| *MYB33* | 13.12 | 51.78 | 0.00 | Proved to facilitate anther development redundantly | Millar and Gubler 2005 |
| *ILR3* | 333.63 | 106.07 | 33.06 | Required to maintain Fe homeostais in correlation with a large amount of Fe3+ in sepals | Sudre et al, 2013 |
| *CUC1* | 8.08 | 2.65 | 0.00 | Responsible to mechanisms to separate organs developing at adjacent positions during early flower development. | Aida et al, 1997 |
| *CUC2* | 17.94 | 17.66 | 7.68 |  |  |
| *IAA9* | 26.10 | 76.06 | 33.05 | Repressors of early auxin response genes at low auxin concentrations. | Wang et al, 2009;  Liscum and Reed, 2002 |
| *IAA27* | 173.21 | 23.97 | 15.68 |  |  |
| *PIN1* | 52.44 | 17.25 | 43.85 | Expression of PIN1 and essential for correct auxin efflux into the early stages of female gametophyte development | Wang et al, 2009 |
| *EIN3* | 161.47 | 45.70 | 13.69 | Delays flowering via repression of the *LFY* and *SOC1* genes | Ceccato et al, 2011 |
| *COL4* | 646.84 | 192.96 | 155.56 | Transcription factor involved in the light input to the circadian clock. | Lee et al, 2010 |
| *COL5* | 1099.61 | 51.63 | 18.13 | Induce flowering in short-day grown Arabidopsis | Hassidim et al, 2009 |
| *COL9* | 8.19 | 2.70 | 0.00 | Delays flowering by reducing expression of CO and FT. | Cheng et al, 2005 |
