# Supplementary material for: Different expression pattern of flowering pathway genes contribute to male or female organ development during floral transition in the monoecious weed Ambrosia artemisiifolia L. (Asteraceae)
Source: PeerJ. 2019 Oct 4;7:e7421. doi: 10.7717/peerj.7421 (PMC6779118; doi:10.7717/peerj.7421)
Supplement: Supplemental Information 10 [file peerj-07-7421-s010.docx]

| **Gene homolog** | **RPKM value (1F)** | **RPKM value (2F)** | **RPKM value (1M)** | **RPKM value (2M)** | **Function** | **References** |
| --- | --- | --- | --- | --- | --- | --- |
| *MYB33* | 17.98 | 23.39 | 233,20 | 228.81 | Proved to facilitate anther development redundantly | Millar and Gubler 2005 |
| *ILR3* | 395.67 | 321.32 | 312.86 | 318.10 | Required to maintain Fe homeostais in correlation with a large amount of Fe3+ in sepals | Sudre et al. 2013 |
| *CUC1* | 29.43 | 22.25 | 37.56 | 40.18 | Responsible to mechanisms to separate organs developing at adjacent positions during early flower development. | Aida et al. 1997 |
| *CUC2* | 6.13 | 0.00 | 10.77 | 5.49 |  |  |
| *IAA9* | 263.80 | 139.43 | 351.46 | 371.83 | Repressors of early auxin response genes at low auxin concentrations. | Wang et al. 2009;  Liscum and Reed. 2002 |
| *IAA27* | 204.66 | 546.24 | 68.02 | 59.06 |  |  |
| *PIN1* | 155.54 | 29.07 | 43.34 | 42.69 | Expression of PIN1 and essential for correct auxin efflux into the early stages of female gametophyte development | Wang et al. 2009 |
| *EIN3* | 97.18 | 134.97 | 254.33 | 230.72 | Delays flowering via repression of the *LFY* and *SOC1* genes | Ceccato et al. 2011 |
| *COL4* | 1478.03 | 1564.22 | 552.40 | 509.76 | Transcription factor involved in the light input to the circadian clock. | Lee et al. 2010 |
| *COL5* | 478.01 | 549.48 | 257.51 | 261.93 | Induce flowering in short-day grown Arabidopsis | Hassidim et al. 2009 |
| *COL9* | 53.41 | 38.76 | 15.38 | 15.62 | Delays flowering by reducing expression of CO and FT. | Cheng et al. 2005 |
