# Supplementary material for: Different expression pattern of flowering pathway genes contribute to male or female organ development during floral transition in the monoecious weed Ambrosia artemisiifolia L. (Asteraceae)
Source: PeerJ. 2019 Oct 4;7:e7421. doi: 10.7717/peerj.7421 (PMC6779118; doi:10.7717/peerj.7421)
Supplement: Supplemental Information 15 [file peerj-07-7421-s015.docx]

|  | **GO-Terms** | **#Seqs** | **Gene name** |
| --- | --- | --- | --- |
| **Biological process** | cellular metabolic process | 38 | PMADS2, OAS, GAI1, SPA, SOC1, SVP, IAA27, CSTF77, BEE1, SEP4, IAA9, SEP2, SEP3, SEP1, FUL, AGL24, CUC2, CUC1, AP3_3, AP1, AP2, PHYB, PHYA, AG, LAC2, EIN3, CSTF64, AP3_2, CAL, AP3_1, VIP5, VIP6, SEP2_2, PI, PBL9, SEP3_2, VIP3, VIP4 |
|  | organic substance metabolic process | 38 | OAS, PMADS2, GAI1, SPA, SOC1, SVP, IAA27, CSTF77, BEE1, SEP4, IAA9, SEP2, SEP3, SEP1, FUL, AGL24, CUC2, CUC1, AP3_3, AP1, AP2, PHYB, PHYA, AG, LAC2, EIN3, CSTF64, AP3_2, CAL, AP3_1, VIP5, VIP6, SEP2_2, PI, PBL9, SEP3_2, VIP3, VIP4 |
|  | primary metabolic process | 37 | PMADS2, OAS, GAI1, SPA, SOC1, SVP, IAA27, CSTF77, BEE1, SEP4, IAA9, SEP2, SEP3, SEP1, FUL, AGL24, CUC2, CUC1, AP3_3, AP1, AP2, PHYB, PHYA, AG, EIN3, CSTF64, AP3_2, CAL, AP3_1, VIP5, VIP6, SEP2_2, PI, PBL9, SEP3_2, VIP3, VIP4 |
|  | nitrogen compound metabolic process | 36 | PMADS2, GAI1, SPA, SOC1, SVP, IAA27, CSTF77, BEE1, SEP4, IAA9, SEP2, SEP3, SEP1, FUL, AGL24, CUC2, CUC1, AP3_3, AP1, AP2, PHYB, PHYA, AG, EIN3, CSTF64, AP3_2, CAL, AP3_1, VIP5, VIP6, SEP2_2, PI, PBL9, SEP3_2, VIP3, VIP4 |
|  | regulation of cellular process | 34 | PMADS2, GAI1, SOC1, SVP, IAA27, CSTF77, BEE1, SEP4, IAA9, SEP2, COP1, SEP3, SEP1, FUL, AGL24, CUC2, CUC1, AP3_3, AP1, AP2, PHYB, PHYA, AG, EIN3, AP3_2, CAL, AP3_1, VIP5, VIP6, SEP2_2, CRY1, PI, PIN1, SEP3_2 |
|  | biosynthetic process | 34 | PMADS2, OAS, GAI1, SOC1, SVP, IAA27, CSTF77, BEE1, SEP4, IAA9, SEP2, SEP3, SEP1, FUL, AGL24, CUC2, CUC1, AP3_3, AP1, AP2, PHYB, PHYA, AG, EIN3, AP3_2, CAL, AP3_1, VIP5, VIP6, SEP2_2, PI, SEP3_2, VIP3, VIP4 |
|  | regulation of metabolic process | 32 | PMADS2, GAI1, SOC1, SVP, IAA27, CSTF77, BEE1, SEP4, IAA9, SEP2, SEP3, SEP1, FUL, AGL24, CUC2, CUC1, AP3_3, AP1, AP2, PHYB, PHYA, AG, EIN3, AP3_2, CAL, AP3_1, VIP5, VIP6, SEP2_2, PI, SEP3_2, VIP3 |
|  | positive regulation of metabolic process | 20 | PMADS2, AP3_3, AP1, AG, SOC1, SVP, AP3_2, CAL, AP3_1, SEP4, VIP5, SEP2, SEP3, SEP2_2, SEP1, PI, SEP3_2, FUL, AGL24, VIP3 |

|  | **GO-Terms** | **#Seqs** | **Gene name** |
| --- | --- | --- | --- |
| **Molecular function** | organic cyclic compound binding | 42 | PMADS2, SPA, CYP450_CYP72A219, SOC1, SVP, CSTF77, MYB33, SEP4, SEP2, SEP3, SEP1, P450_86B1_like, WIP2, FUL, AGL24, MYB35, CUC2, CUC1, AP3_3, SPL3, AP1, AP2, SPL1, AG, MYB80, LMI2, EIN3, CSTF64, AP3_2, SUP, CAL, AP3_1, VIP5, MYB44, SEP2_2, VRN1, MYB61, PI, PBL9, MYB5, SEP3_2, MYB26 |
|  | heterocyclic compound binding | 42 | PMADS2, SPA, CYP450_CYP72A219, SOC1, SVP, CSTF77, MYB33, SEP4, SEP2, SEP3, SEP1, P450_86B1_like, WIP2, FUL, AGL24, MYB35, CUC2, CUC1, AP3_3, SPL3, AP1, AP2, SPL1, AG, MYB80, LMI2, EIN3, CSTF64, AP3_2, SUP, CAL, AP3_1, VIP5, MYB44, SEP2_2, VRN1, MYB61, PI, PBL9, MYB5, SEP3_2, MYB26 |
|  | protein binding | 22 | PMADS2, AP3_3, AP1, PHYB, PHYA, AG, ILR3, SOC1, SVP, AP3_2, CAL, AP3_1, SEP4, BEE1, SEP2, SEP3, SEP2_2, SEP1, PI, SEP3_2, FUL, AGL24 |
|  | DNA-binding transcription factor activity | 20 | PMADS2, AP3_3, AP1, AP2, AG, EIN3, SOC1, SVP, AP3_2, CAL, AP3_1, SEP4, SEP2, SEP3, SEP2_2, SEP1, PI, SEP3_2, FUL, AGL24 |
|  | ion binding | 18 | Seq79, SPL3, GA2ox8, GA2ox1, SPL1, SPA, ACA7, COL9, GA2ox3, LAC2, COL4, CYP450_CYP72A219, CO, FT, TFL1, PBL9, P450_86B1_like, GA3ox1 |
|  | oxidoreductase activity | 7 | GA2ox8, GA2ox1, GA2ox3, LAC2, P450_86B1_like, GA3ox1, CYP450_CYP72A219 |

|  | **GO-Terms** | **#Seqs** |  |
| --- | --- | --- | --- |
| **Cellular component** | intracellular organelle | 45 | PMADS2, SYN_112, GAI1, COL9, COL4, SOC1, SVP, IAA27, CSTF77, SEP4, MYB33, SEP2, IAA9, SEP3, SEP1, FUL, AGL24, MYB35, CUC2, CUC1, Seq79, AP3_3, SPL3, AP1, AP2, SPL1, AG, MYB80, LMI2, EIN3, CO, AP3_2, CAL, AP3_1, MYB44, VIP5, SEP2_2, VRN1, MYB61, PI, SEP3_2, MYB5, MYB26, VIP3, VIP4 |
|  | membrane-bounded organelle | 45 | PMADS2, SYN_112, GAI1, COL9, COL4, SOC1, SVP, IAA27, CSTF77, SEP4, MYB33, SEP2, IAA9, SEP3, SEP1, FUL, AGL24, MYB35, CUC2, CUC1, Seq79, AP3_3, SPL3, AP1, AP2, SPL1, AG, MYB80, LMI2, EIN3, CO, AP3_2, CAL, AP3_1, MYB44, VIP5, SEP2_2, VRN1, MYB61, PI, SEP3_2, MYB5, MYB26, VIP3, VIP4 |
|  | intracellular | 45 | PMADS2, SYN_112, GAI1, COL9, COL4, SOC1, SVP, IAA27, CSTF77, SEP4, MYB33, SEP2, IAA9, SEP3, SEP1, FUL, AGL24, MYB35, CUC2, CUC1, Seq79, AP3_3, SPL3, AP1, AP2, SPL1, AG, MYB80, LMI2, EIN3, CO, AP3_2, CAL, AP3_1, MYB44, VIP5, SEP2_2, VRN1, MYB61, PI, SEP3_2, MYB5, MYB26, VIP3, VIP4 |
|  | intracellular part | 45 | PMADS2, SYN_112, GAI1, COL9, COL4, SOC1, SVP, IAA27, CSTF77, SEP4, MYB33, SEP2, IAA9, SEP3, SEP1, FUL, AGL24, MYB35, CUC2, CUC1, Seq79, AP3_3, SPL3, AP1, AP2, SPL1, AG, MYB80, LMI2, EIN3, CO, AP3_2, CAL, AP3_1, MYB44, VIP5, SEP2_2, VRN1, MYB61, PI, SEP3_2, MYB5, MYB26, VIP3, VIP4 |
|  | intrinsic component of membrane | 7 | SYN_112, TET8, GDSL2, NIP, PIN1, P450_86B1_like, CYP450_CYP72A219 |
